# BioMet Toolbox 2.0: genome-wide analysis of metabolism and omics data

**DOI:** 10.1093/nar/gku371

**Published:** 2014-05-03

**Authors:** Manuel Garcia-Albornoz, Subazini Thankaswamy-Kosalai, Avlant Nilsson, Leif Väremo, Intawat Nookaew, Jens Nielsen

**Affiliations:** Department of Chemical and Biological Engineering, Chalmers University of Technology, Göteborg, Sweden

## Abstract

Analysis of large data sets using computational and mathematical tools have become a central part of biological sciences. Large amounts of data are being generated each year from different biological research fields leading to a constant development of software and algorithms aimed to deal with the increasing creation of information. The BioMet Toolbox 2.0 integrates a number of functionalities in a user-friendly environment enabling the user to work with biological data in a web interface. The unique and distinguishing feature of the BioMet Toolbox 2.0 is to provide a web user interface to tools for metabolic pathways and omics analysis developed under different platform-dependent environments enabling easy access to these computational tools.

## INTRODUCTION

In the last few years, computer sciences and mathematics have contributed to the development of new strategies applied to biological research. Several free-access databases along with tools and software packages are available online aiming for the extraction of valuable information from raw data (1). In the field of biological sciences the use of computational tools has enabled a rapid expansion of new applications for data analysis and *in silico* simulations. New algorithms and software tools are being constantly developed helping to deal with the explosively expanding amount of data produced by science and industry (1,2).

Systems biology has been described as the holistic analysis of biological systems with the primary goal to understand the interactions between different components and their regulation. Genome-scale metabolic models (GEMs) and gene expression profiles are valuable sources of information for gene–gene interaction and phenotype predictions applied to systems biology (3,4).

The reconstruction and modification of GEMs and omics data analysis are tasks that require specialized software tools (4,5). Several tools for analysis, simulation, editing, running and visualization of GEMs and omics data have been developed and are already available; however, most of the new software and programs generated for bioinformatics applications require the installation of libraries or other additional software packages before their use. In addition to this, some programs require programming knowledge on a command-based platform, which can be bothersome for an inexperienced user. To overcome these difficulties, the upgraded version of the BioMet Toolbox (6) is, therefore, intended to provide a web user interface (WUI) to platform-dependent tools enabling the access by unexperienced users to these computational tools.

## FEATURES

**Figure 1.**
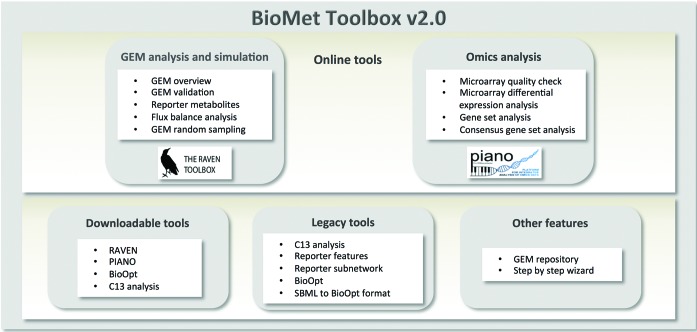
BioMet Toolbox v2.0 organization and functionalities.

The main contribution of the BioMet Toolbox 2.0, is the online WUI for the previously developed RAVEN (4) and PIANO (3) tools. RAVEN is a software for GEM analysis and simulation developed in MATLAB. PIANO is a software developed in R for omics data analysis. The WUI to these platform-dependent tools provided by the BioMet Toolbox 2.0 enables the use of their functions under a user-friendly environment with no necessity of previous platform knowledge. The BioMet Toolbox v2.0 web site has been written in PHP, HTML and JavaScript. RAVEN can also be downloaded directly from the BioMet Toolbox 2.0 web site including installation guide and tutorial and PIANO can be downloaded from Bioconductor (7) through the provided link. In addition to the online WUI tools, the BioMet Toolbox 2.0 includes improvements to the user interface with a more logical layout, an expanded collection of high quality GEMs of different organisms (GEM repository) and a collection of legacy tools from the previous version of the BioMet Toolbox (Figure 1).

**Figure 2.**
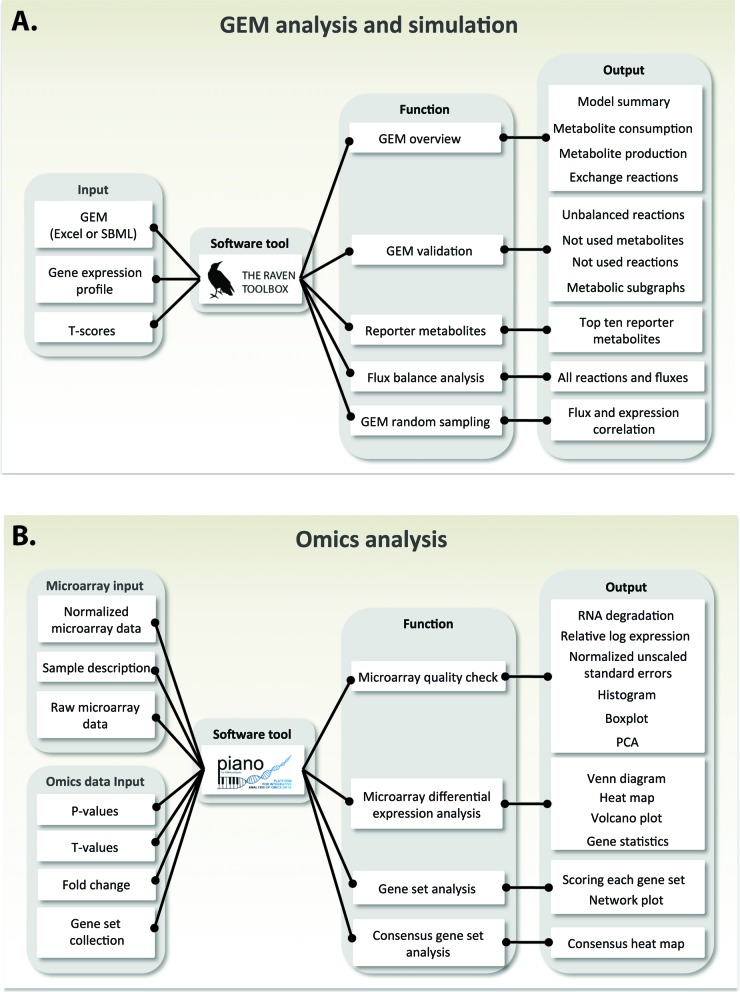
Overview of the online tools workflow. Information flows through the model from left to right with each column showing the corresponding levels. (**A**) Online tool for GEM analysis and simulation. (**B**) Online tool for omics analysis.

The provided online WUI tools in BioMet Toolbox v2.0 offer two major groups of analysis which are: (i) GEM analysis and simulation and (ii) omics data analysis. The analysis and simulation of GEMs through the WUI tool (RAVEN powered) provide functionalities as: (i) GEM overview, (ii) GEM validation, (iii) Reporter metabolites, (iv) Flux balance analysis (FBA) and (v) GEM random sampling (Figure 2A). For the GEM overview, users are allowed to upload their GEM in specific Excel format or SBML format (8) together with omics data to check the entities of the uploaded GEM, such as number of genes, reactions and metabolites for each compartment, the reactions involving only products or only reactants and the metabolites that can be produced and consumed. In GEM validation the unbalanced reactions (Elemental balance), dead end reactions, dead end metabolites, connectivity and isolated networks can be evaluated and queried before going to the next step. For GEM analysis FBA simulations and integrative analysis of omics data using GEM as a scaffold, such as Reporter metabolite analysis (9,10), and the identification of transcriptional flux regulation using random sampling (11), are included.

Statistical values derived from omics data can be uploaded and analyzed through the WUI of omics analysis (PIANO powered), providing functionalities as: (i) Microarray quality check, (ii) Microarray differential expression analysis, (iii) Gene set analysis (GSA) and (iv) Consensus gene set analysis (Figure 2B). Since microarray data are widely used and shared in the research community, BioMet Toolbox 2.0 provides a standard microarray analysis work flow including quality assessment, normalization and differential expression analysis. Each analysis will generate result tables and appropriate plots which can be viewed directly in the WUI or be downloaded by the user. The Gene set analysis function collects a number of GSA methods into the same platform, making it easier to test different methods using the same settings, format and input. The input to this tool is a collection of gene sets and gene-level statistics. The gene sets can be, e.g. Gene Ontology terms (12) or any other terms, enabling the identification of statistically significant biological processes. The gene-level statistics can be, e.g. *P*-values and *t*-values from the Microarray differential expression analysis module or statistical values from RNA-seq data or other gene-centered omics data. The output of this tool is a network plot detailing the biological functions, and their connections, that are affected by differentially expressed genes along with a table in Excel format with the number of genes in each gene set, the gene set statistics and their *P*-values (normal and adjusted). The Consensus gene set analysis allows the user to combine results from different gene set analyses and is performed under a combination of different GSA methods in order to obtain a consensus heat map as an output.

Additionally to the WUI tools, the BioMet Toolbox 2.0 includes an expanded collection of GEMs (Models repository) including several high quality GEM reconstructions for different organisms. For fungi the available models are: *Saccharomyces cerevisiae*, *Pichia pastoris*, *Pichia stipitis*, *Aspergillus niger*, *Aspergillus oryzae* and *Aspergillus nidulans*. For bacteria the available models are: *Streptomyces coelicolor*, *Lactococcus lactis*, *Synechocystis* sp. PCC6803 and *Amycolatopsis balhimycina*. All the models in the GEM repository are available in several formats. Submission of new GEMs is allowed and highly encouraged in order to expand the GEM database.

Several web sites are available for either GEM analysis or omics data analysis (13–19). Nevertheless, as outlined above, the BioMet Tolbox 2.0 offers an expanded selection of functions and tools all in one place enabling the user to combine the results of GEM analysis and omics data analysis.

Along with the WUI tools, some legacy tools (C13, BioOpt, Reporter Features and Reporter Subnetwork) from the previous version of the BioMet Toolbox (6) are still accessible from the BioMet Toolbox 2.0.

## SHOW CASE

To illustrate the use of the BioMet Toolbox 2.0 some *in silico* simulations were performed by using the RAVEN powered online tools. These simulations were done by first uploading a yeast model (yeast 5.32) (20). The simulated media was a minimal chemostat medium (glucose-limited) under aerobic and anaerobic condition with a constrained uptake rate for glucose specified from a condition of the previously reported *in vivo* values (21). Each model file (aerobic and anaerobic) was then uploaded to the BioMet Toolbox v2.0 for maximization of biomass production as an objective function. The GEM overview and GEM validation options were run in order to find any error in the model (Table 1A). These tools returned a summary of 897 genes, 2034 reactions and 1600 metabolites.

**Table 1.** (A) Part of the Script progress output for GEM overview showing the number of reactions, metabolites and genes for simulated minimal chemostat medium under aerobic condition. (B) Random sampling output for the GEM's first top 20 reactions sorted by the highest probabilities of change in flux and expression in the sam direction between two conditions (aerobic and anaerobic).

A	B					
GEM characteristics		Reaction name	Change both in flux and expression in the same direction	Change in expression but not in flux	Change in flux but not in expression
id	'ymn5_0'				
description	'Yeast metabolic network'				
annotation	[1×1 struct]	phosphoglycerate kinase	0.6473	0	0.3527	
rxns	{2034×1 cell}	triose-phosphate isomerase	0.6420	0.0008	0.3567
mets	{1600×1 cell}	H+ diffusion	0.6164	0.0071	0.3722
comps	{16×1 cell}	enolase	0.6156	0	0.3844
compNames	{16×1 cell}	glucose-6-phosphate isomerase	0.5796	0	0.4204	
compOutside	{16×1 cell}	D-lactate/pyruvate antiport	0.5664	0	0.4336
rxnNames	{2034×1 cell}	D-lactate transport	0.5653	0	0.4347	
rxnComps	[2034×1 double]	ammonia transport	0.5526	0.0886	0.3092
grRules	{2034×1 cell}	oxygen exchange	0.5444	0.0002	0.4553
rxnGeneMat	[2034×897 double]	phosphoglycerate mutase	0.5210	0	0.4790
subSystems	{2034×1 cell}	glycine h-methyltransferase	0.5029	0.0531	0.4016
eccodes	{2034×1 cell}	fructose-bisphosphate aldolase	0.4839	0	0.5159	
genes	{897×1 cell}	bicarbonate transport	0.4812	0.0056	0.5047
geneComps	[897×1 double]	bicarbonate formation	0.4711	0.0056	0.5074
metNames	{1600×1 cell}	glutamate dehydrogenase (NAD)	0.4508	0.1088	0.3412
metComps	[1600×1 double]	pi-oribosyl imidazole. synth.	0.4506	0.2054	0.2361
inchis	{1600×1 cell}	pi-oribosylglycinamidine synth.	0.4421	0.2054	0..363
metFormulas	{1600×1 cell}	ammonium exchange	0.4385	0.0709	0.4197
metMiriams	{1600×1 cell}	pantothenate transport	0.4385	0.1894	0.2598
unconstrained	[1600×1 double]	tyrosine transport	0.4360	0.1665	0.2877

The shift from respiratory to fermentative metabolism leading to ethanol production was further investigated using the GEM as a scaffold for integrative analysis of transcriptome data from these growth conditions (22). The transcriptome data were retrieved from the Array express number E-MEXP-3704. GEM random sampling was run, comparing the significance of change in flux between aerobic and anaerobic conditions (Table 1B). This tool generates a *N* × 3 column matrix with the probabilities of a reaction: (i) changing both in flux and expression in the same direction, (ii) changing in expression but not in flux and (iii) changing in flux but not in expression or changing in opposed directions in flux and expression. A comparison between simulated productions, uptakes and growth rates and those obtained from previously published experimental *in vivo* results for aerobic and anaerobic conditions (21) are shown in Table 2A. The same model file was then uploaded along with the *P-values* from differential gene expression analysis under the two conditions to identify metabolite hotspots. The top 10 ranking metabolites are presented in Table 2B. Clearly, components associated with respiration and adenosine triphosphate generation (including proton in the mitochondria) are among the top-reporter metabolites as the energy generation is completely shifted from respiration to fermentation when changing from aerobic to anaerobic growth.

**Table 2.** (A) Comparison of previously reported *in vivo* fluxes and growth rates on aerobic and anaerobic conditions. Uptake and excretion fluxes are reported in mmol/g dry weight/h and growth rates in h - 1. Glucose was used as the limiting substrate. (B) Top 10 most significant reporter metabolites obtained from the reporter metabolites analysis for *S. cerevisiae* on minimal chemostat medium while changing from aerobic to anaerobic growth.

A		
		BioMet Toolbox v2
	*In vivo*	*In silico* simulation
***Aerobic***		
Carbon limited aerobic glucose uptake	1.15	1
Carbon limited aerobic oxygen uptake	2.7	2.74
Carbon limited aerobic CO_2_ excretion	2.8	2.78
Carbon limited aerobic ethanol production	0	0
Carbon limited aerobic growth rate	0.1	0.09
***Anaerobic***		
Carbon limited anaerobic glucose uptake	2.3	2
Carbon limited anaerobic oxygen uptake	0	0
Carbon limited anaerobic CO_2_ excretion	3.8	3.4
Carbon limited anaerobic ethanol production	3	3.4
Carbon limited anaerobic growth rate	0.03	0.04
**B**				
Top 10 Reporter metabolites		
ferricytochrome c [mitochondrion]	ADP [mitochondrion]	
ferrocytochrome c [mitochondrion]	H+ [mitochondrion]	
ubiquinone-6 [mitochondrion]	urea [cytoplasm]	
ubiquinol-6 [mitochondrion]	allantoate [cytoplasm]	
phosphate [mitochondrion]	L-cysteine [extracellular]	

**Figure 3.**
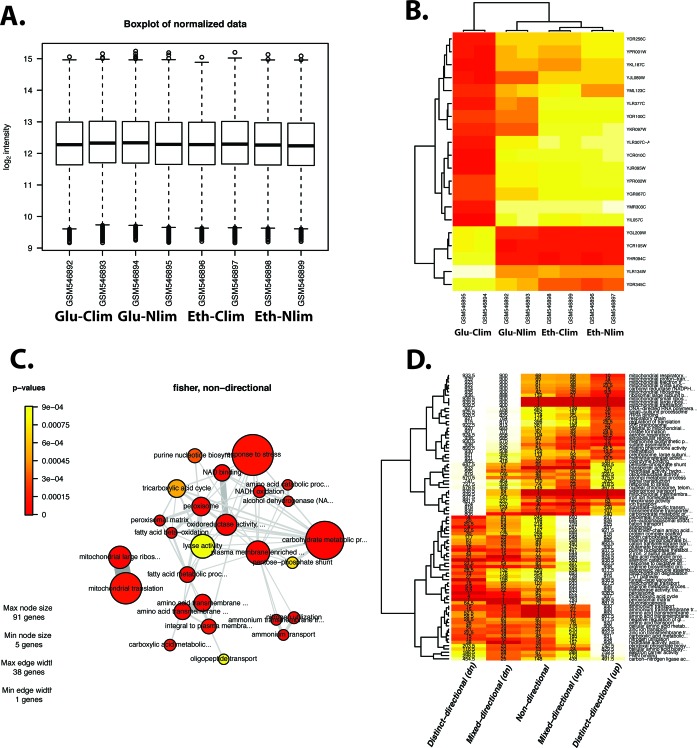
Output results from the Omics analysis online tool. (**A**) Boxplot of normalized data from the Microarray quality check option. (**B**) Heatmap from the Microarray differential expression analysis option. (**C**) Gene set analysis. (**D**) Consensus gene set analysis

For the PIANO powered online tools a gene expression data set from *Saccharomyces cerevisiae* was downloaded from the Gene Expression Omnibus Database using accession number GSE21988 containing the nutrient-dependent regulation gene expression in *S. cerevisiae*. This data set contains the gene expression profiles from *S. cerevisiae* while growing in chemostat cultures on carbon or nitrogen starvation using either glucose or ethanol as carbon source (23). For this experiment, growth limitation was done by either carbon or nitrogen. When carbon was limited, the growth was tested on either glucose or ethanol (using ammonium sulfate as the nitrogen source). When ammonium sulfate was the limited factor, either glucose or ethanol was used as the carbon source. Raw .CEL files were uploaded to the online tool for omics analysis and the Microarray quality check was first performed, obtaining all the plots for raw and normalized data (See example in Figure 3A). For the Microarray differential expression analysis the compared conditions were: Glucose versus Carbon limited, Glucose versus Nitrogen limited, Ethanol versus Carbon limited and Ethanol versus Nitrogen limited. The heatmap obtained can be observed in Figure 3B. The heatmap shows the expression levels of the top significant differentially expressed genes for the compared conditions. In this chart the differences between the four conditions are clearly observed. The results from gene set analysis were illustrated as a network plot in Figure 3C detailing the biological functions enriched with significantly differentially expressed genes. Furthermore the results from the consensus gene set analysis are illustrated in Figure 3D as a heatmap and provides similar information as the network plot in the GSA, but by showing the directionality of the gene set (up or down regulated) represents better detail for further biological interpretation.

These examples show how the BioMet Toolbox v2.0 can be used to obtain quantitative estimations of fluxes and growth rates in good agreement with previously reported *in vivo* results. At the same time, this tool provides a helpful and robust way to perform analysis from omics data, which can be used to identify new metabolic routes, gene targets for genetic engineering and transcriptional changes occurring in biological systems.

## SUMMARY

The BioMet Toolbox v2.0 offers a selection of online software tools for biological data analysis along with free access to a collection of GEMs for their use in phenotype simulations. Among the advantages of using the BioMet Toolbox v2.0 are its web-based free availability and its user-friendly and platform-independent online tools allowing for omics data analysis and GEM analysis and simulation under a WUI environment, suitable for both inexperienced and advanced users. The online interface for the RAVEN and PIANO powered tools represents an important advance in the field of system biology allowing the final user to perform different features from an easy to use WUI environment avoiding any complicated software installation. The BioMet Toolbox v2.0 offers the alternative option to download the RAVEN and PIANO in order to perform a wider range of functionalities under different command-based platforms. Additionally, the BioMet Toolbox v2.0 offers the possibility of constant growth in additional features and updated functionalities.

## AVAILABILITY

BioMet Toolbox v2.0 is freely available at www.biomet-toolbox.org. Contact: biomet2 [at] sysbio.se.

## FUNDING

European Research Council (INSYSBIO) [247013]; Chalmers Foundation; Knut and Alice Wallenberg Foundation; Bioinformatics Infrastructure for Life Sciences (BILS) and Samsung Electronics Inc. Source of open access funding: Chalmers Library.

*Conflict of interest statement*. None declared.
